# The Molecular Study of Antibiotic Resistance to Quinolones in *Salmonella enterica* Strains Isolated in Tehran, Iran

**DOI:** 10.2174/1874285801711010189

**Published:** 2017-09-21

**Authors:** Shirin Malehmir, Reza Ranjbar, Naser Harzandi

**Affiliations:** 1 Department of Microbiology, Karaj Branch, Islamic Azad University, Karaj, Iran; 2 Molecular Biology Research Center, Baqiyatallah University of Medical Sciences, Tehran, Iran

**Keywords:** Nalidixic acid resistance, *Qnr*, *Salmonella enterica*, Quinolone, *QnrA*, CLSI

## Abstract

**Introduction:**

*Salmonella* is known as one of the most important causes of gastrointestinal disease in the world. Quinolones and fluoroquinolones are used successfully in the treatment of salmonellosis particularly for infections that have become resistant to several antibiotics. But non-susceptible isolates to quinolones have been reported in several countries. The data are limited about the prevalence of quinolone-resistant isolates in our country. Therefore, this study investigated the plasmid-mediated quinolone resistance genes in *Salmonella enterica* isolated in Children's Medical Center in Tehran during 2014-2015.

**Methods and Materials:**

*Salmonella* isolates were isolated and identified using standard microbiological methods. Antibiotic susceptibility testing and screening of *Salmonella* strains resistant to quinolones were performed according to the CLSI guidelines. The molecular investigation was done using specific primers for detection of qnr genes including: *qnr*A, *qnr*B and *qnr*S, by polymerase chain reaction.

**Results:**

Overall, 92 (66.6%) strains were resistant to nalidixic acid. None of the strains showed resistance to ciprofloxacin. Out of the 92 nalidixic acid resistant strains, 52 (56.52%) harbored *qnr*S genes, 15 strains (16.30%) had both *qnr*A and *qnr*S genes. Two (1.1%) isolates were positive for *qnr*B gene. Twenty four (26.08%) nalidixic acid resistant isolates did not have any qnr qens.

**Conclusion:**

The results of this study show high prevalence of resistance to nalidixic and qnr genes in *Salmonella* isolates. Plasmid nature of this type of resistance poses an increased risk of dissemination of quinolone resistance between *Salmonella* and non-*Salmonella* isolates circulating in hospitals environments.

## 1 INTRODUCTION

The infections caused by enteric pathogens comprise second commonest medical problems after respiratory infectious disease [1]. *Salmonella*, is among the most prevalent and endemic food borne pathogens in Iran [2].

*Salmonella* is one of the most important genera of Enterobacteriaceae, causing a wide variety of different diseases in society. In Kaufmann-White classification, each serotype is considered a genus based on the antigenic lipopolysaccharide (LPS) structure of cell surface (AgO) and new proteins (AgH), accordingly, 2,600 genus was suggested for this organism [3-6].

Over 90% of serotypes causing intestinal illness in humans are related to *Salmonella typhi*, *S. typhimurium*, *S. enteritidis* and *S. infantis* [7]. Usually recovery from non typhoidal disease can be achieved without antibiotics. The antibiotics are used in patients suffering enteric fever, septicemia and also in high risk persons, including patients with a weak immune system, malnutrition and malignancy. Ampicillin, chloramphenicol and co-trimoxazole, have been considered for the treatment of salmonellosis; however due to increased resistance of these compounds, usage of fluoroquinolones such as ciprofloxacin and cephalosporin is increased in adults and children [7, 8]. Fluoroquinolones are broad-spectrum antimicrobial drugs which are used for the treatment of clinical salmonellosis infections in elderly or immunocompromised patients as well as patients suffering enteric fever, invasive disease or long-term carriers of *Salmonella* [8-10].

The increase of antibiotic resistance in developing countries’ patients has become an important concern [11]. Acquisition of antibiotic resistance occurs through several mechanisms [12] including chromosomal mutation, resistance transfer to other bacteria through the transposable elements such as plasmids and transposons, decrease in the cell wall permeability of antibiotics, antibiotics enzymatic inactivation, and efflux pump mechanisms for removing antibiotics [13].

Rapid detection and molecular epidemiology investigation of resistance genes in bacteria are extremely important [14-17].

We aimed to investigate the plasmid-mediated quinolone resistance genes in *Salmonella enterica* isolated from Children's Medical Center in Tehran during 2014-2015.

## 2 MATERIALS AND METHODS

The study was conducted on clinical specimens recovered from patients who were suspected to *Salmonella* infection admitted to Children's Medical Center in Tehran during the years 2014-2015. Bacterial isolation and identification were done according to the standard microbiological and biochemical methods.

After performing biochemical tests for *Salmonella* confirmation, serotyping was performed to determine the O and H antigens with specific antiserum.

Antibiotic susceptibility testing for nalidixic acid (NA 30μg) and ciprofloxacin (CP 5μg) was done by modified Kirby-Bauer method. Escherichia coli ATCC 25922 were used as a control in the experiment.

DNA was extracted by boiling method. The primers used in this study are listed in Table (**1**) [18]. PCR was performed for the amplification of *qnr*A, *qnr*B, *qnr*S genes in thermocycler machine under the conditions shown in (Table **2**).

## 3 RESULTS

Antibiotic susceptibility testing results showed that 92(66.6%) strains of *Salmonella enterica* were resistant to nalidixic acid. None of the investigated strains showed resistance to ciprofloxacin.

PCR amplified the expected amplicons with sizes of 417bp, 516bp, and 526bp for *qnrS, qnrA* and *qnrB* genes, respectively. Fig. (**1**) shows amplified target genes from representatives *Salmonella* isolates harboring different *qnr* genes. Overall, out of 92 nalidixic acid resistant *Salmonella* strains, 52 (56.5%) had *qnr*S gene. The genes *qnr*A and *qnr* B were observed in 28 and 2 strains respectively. Among nalidixic acid-resistant strains, 15 (16.30%) strains harbored *qnr*S gene together. Only one strain contained *qnr* B gene.

## 4 DISCUSSION

In the past, Ampicillin, chloramphenicol and co-trimoxazole, have been used for the treatment of salmonellosis, but today, fluoroquinolones such as ciprofloxacin and cephalosporin are used for adults and children respectively [8]. The strains resistant to nalidixic acid have a reduced sensitivity to fluoroquinolones [18, 19].

In recent years, several cases of treatment failure with fluoroquinolones have been reported in patients infected by *Salmonella* strains resistant to nalidixic acid and reduced sensitivity to ciprofloxacin. Unfortunately, their number is increasing [20, 21].

In Nigeria, Akinyemi *et al.* showed that 42%, 43%, 42%, 47%, 51%, 57%, 56% and 59% of *Salmonella* strains recovered in the years 1997-2004 were resistant to nalidixic acid respectively [22].

Similar results were reported by Ghaderi *et al.* in which 67.2% *Salmonella* strains were resistant to nalidixic acid [4].

In a study conducted in Spain by Prats *et al*. showed 10% increase in resistance of nalidixic acid from 0.1% to 11% over seven years [23].

Compared to previous studies, our results show the increasing rates of *Salmonella* strains resistant to nalidixic acid in Tehran. The rate of resistance to nalidixic acid can be important, because fluoroquinolones have been used as a chosen antibiotic in the treatment of infections caused by *Salmonella*. Antibiotic therapy using fluoroquinolones, may not be successful in people who were infected by nalidixic acid resistant *Salmonella* strains [20, 24].

Ferrari *et al*. showed plasmid- depended quinolone resistance, by gene qnr and gene aac (6 ') Ib-cr in 126 *Salmonella* strains isolated from poultry and humans food sources during the years 1999 to 2007 in the state of Paraná (Brazil). Overall, 88% and 23% of strains showed resistant to Nalidixic acid and reduced susceptibility to ciprofloxacin respectively. QnrA1 gene was shown in a *S. enteritidis* strain [25].

Ghaima *et al*. in 2016, accomplished a study to determine the presence of qnr genes in 40 strains of *Salmonella* isolated from stool samples of patients in Iraq. They found 30 isolates (75%) had multidrug-resistant (MDR). Based on the results of PCR, 15 (37.5%), 22 (55%), and 11 (27.5%) isolates had *qnr*B, *qnr*S and qnrA respectively. 10 cases (25%) contain qnrA, qnrB and qnrS [26].

Lunn *et al*. determined the mechanism of plasmid- depended quinolone resistance in 41 collected *Salmonella* strains in 2010. None of the isolates were resistant to ciprofloxacin, while 41.5% of the strains showed resistance to nalidixic acid by mutation in *gyr*A. Although *qep*A, *qnr*B, *qnr*S1, were found in 4 isolates, but the expression of this gene was not accompanied by reduced susceptibility to quinolones [27].

## CONCLUSION

Compared to previous studies, the rate of resistance to nalidixic acid is increasing among clinical strains of *Salmonella enterica* that poses a warning alarm for antibiotic therapy of infections caused by this organism in Iran. The molecular method used in this study was able to show the genetic nature of resistance to quinolones among *Salmonella* strains under the study. Plasmid nature of this type of resistance poses an increased risk of dissemination of quinolone resistance between *Salmonella* and non- *Salmonella* isolates circulating in hospitals environments in our country.

## Figures and Tables

**Fig. (1):**
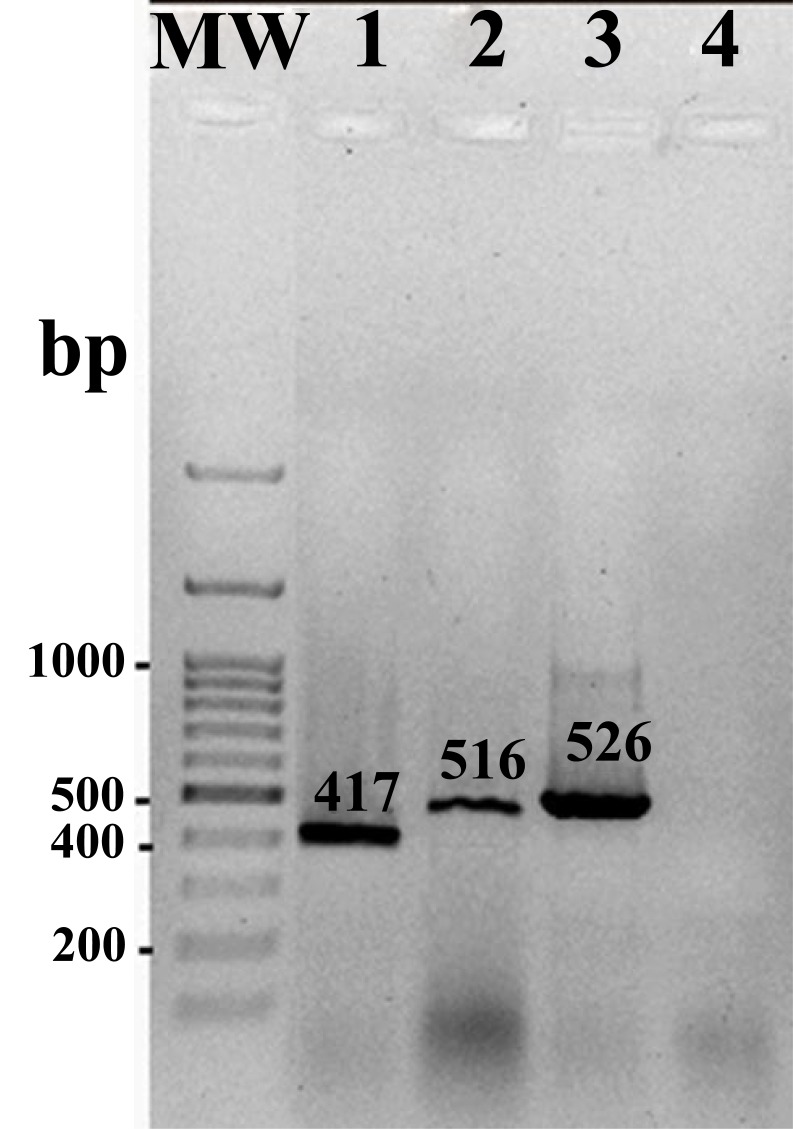
Detection of three genes *qnr*S and *qnr*A and *qnr*B in representative strains of *Salmonella enterica* isolated from clinical specimens. Well 1: a *qnrS* positive isolate. Well 2: one of the clinical isolates with qnrA. Well 3: one of the clinical isolate with gene qnrB. MW: molecular markers (DNA Ladder 100 bp).

**Table 1:** Primers used for detection of *qnr*A, *qnr*B, *qnr*S and their characteristics.

**Amplicon Size (bp)**	**Length**	**Tm** **(C°)**	**Sequences**	**Primers**	**Gene**
516	20	53.9	F: 5′-ATT TCT CAC GCC AGG ATT TG-3′	qnrA	*qnr*A
	20	53.9	R: 5′-GAT CGG CAA AGG TTA GGT CA-3′		
526	22	55.6	F: 5′-GTT GGC GAA AAA ATT GAC AGA A-3′	qnrB	*qnr*B
	20	53.6	R: 5′-ACT CCG AAT TGG TCA GAT CG-3′		
417	20	53.7	F: 5′-ACG ACA TTC GTC AAC TGC AA-3′	qnrS	*qnr*S
	20	54	R: 5′-TTA ATT GGC ACC CTG TAG GC-3′		

**Table 2:** PCR condition for detection of *qnr*A, *qnr*B and *qnr*S genes.

PCR Steps	Temperature C °	Time (min)	Number of Cycles
Initial Denaturation	94	10:00	1
Denaturation	94	1:00	30
Annealing	52	1:00	30
Elongation	72	1:00	30
Final Extension	72	7:00	1
